# Impact of Exogenous dsRNA on miRNA Composition in *Arabidopsis thaliana*

**DOI:** 10.3390/plants13162335

**Published:** 2024-08-22

**Authors:** Nikolay N. Nityagovsky, Konstantin V. Kiselev, Andrey R. Suprun, Alexandra S. Dubrovina

**Affiliations:** Laboratory of Biotechnology, Federal Scientific Center of the East Asia Terrestrial Biodiversity, Far Eastern Branch of the Russian Academy of Sciences, Vladivostok 690022, Russia; nityagovskii@biosoil.ru (N.N.N.); suprun@biosoil.ru (A.R.S.)

**Keywords:** exogenous dsRNA, miRNA, gene silencing, RNA interference, chalcone synthase, plant gene regulation

## Abstract

The application of double-stranded RNAs (dsRNAs) to plant surfaces has emerged as a promising tool for manipulating gene expression in plants and pathogens, offering new opportunities for crop improvement. While research has shown the capability of exogenous dsRNAs to silence genes, the full spectrum of their impact, particularly on the intricate network of microRNAs (miRNAs), remains largely unexplored. Here, we show that the exogenous application of chalcone synthase (*CHS*)-encoding dsRNA to the rosette leaves of *Arabidopsis thaliana* induced extensive alterations in the miRNA profile, while non-specific bacterial neomycin phosphotransferase II (*NPTII*) dsRNA had a minimal effect. Two days after treatment, we detected 60 differentially expressed miRNAs among the 428 miRNAs found in the *A. thaliana* genome. A total of 59 miRNAs were significantly changed after *AtCHS*-dsRNA treatment compared with water and *NPTII*-dsRNA, and 1 miRNA was significantly changed after *AtCHS*-dsRNA and *NPTII*-dsRNA compared with the water control. A comprehensive functional enrichment analysis revealed 17 major GO categories enriched among the genes potentially targeted by the up- and downregulated miRNAs. These categories included processes such as aromatic compound biosynthesis (a pathway directly related to *CHS* activity), heterocycle biosynthesis, RNA metabolism and biosynthesis, DNA transcription, and plant development. Several predicted targets of upregulated and downregulated miRNAs, including *APETALA2*, *SCL27*, *SOD1*, *GRF1*, *AGO2*, *PHB*, and *PHV*, were verified by qRT-PCR. The analysis showed a negative correlation between the expression of miRNAs and the expression of their predicted targets. Thus, exogenous plant gene-specific dsRNAs induce substantial changes in the plant miRNA composition, ultimately affecting the expression of a wide range of genes. These findings have profound implications for our understanding of the effects of exogenously induced RNA interference, which can have broader effects beyond targeted mRNA degradation, affecting the expression of other genes through miRNA regulation.

## 1. Introduction

Spray-induced gene silencing (SIGS), also known as exogenous RNA interference (exo-RNAi), is a novel approach in plant science that utilizes externally applied RNA molecules to manipulate plant traits [1,2,3,4,5]. In this method, plant surfaces are sprayed with solutions containing double-stranded RNA (dsRNA) or short-interfering RNA (siRNA). These RNA molecules act like tiny blueprints that instruct the plant’s RNA interference (RNAi) machinery to degrade specific mRNA molecules, effectively silencing the targeted genes.

The most promising application of SIGS lies in plant fungal and virus protection [5,6,7]. By delivering dsRNAs designed to match the virulence genes in attacking pathogens or plant viruses, researchers can achieve targeted silencing, essentially disarming the invaders. This approach offers a significant advantage over traditional methods such as chemical pesticides, as it is specific and avoids harming beneficial organisms or leaving harmful substances. SIGS is considered a potential alternative to genetically modified (GM) plants, addressing public concerns surrounding GMOs [5,8]. 

Beyond disease control, SIGS holds immense potential for tailoring plant traits. Researchers have successfully used exogenous dsRNAs to silence specific plant genes and observed the resulting biochemical and phenotypic changes in the plant [9,10,11,12,13,14,15]. This approach has provided opportunities to understand the role of individual plant genes in plant development [9], metabolism [10,11,12,13], and the stress response [14,15]. While research in this area is still emerging, several studies have shown promise, revealing that SIGS can be used to have an effect on plants and change their properties over a certain period of time. These studies applied dsRNAs targeting specific genes in Arabidopsis, tobacco, amaranth, orchid, tomato, and grapevine. The results showed a decrease in the targeted gene’s mRNA levels, indicating successful gene silencing. The exogenously induced silencing of plant endogenous genes translated into desirable changes in the plants, such as altered flower morphology [9], improved resistance to downy mildew [14], enhanced tolerance to drought stress [15], and anthocyanin biosynthesis [11,12,13]. Researchers are also exploring novel delivery methods for dsRNAs to target plant endogenes, such as nanoparticle carriers [16,17] and laser-assisted treatments [18], to improve the dsRNA uptake and efficacy within plant cells. 

In our recent study, we demonstrated that the silencing of genes involved in anthocyanin production was demonstrated in *Arabidopsis thaliana* [11,13] and *Solanum lycopersicum* [12]. Derived from the phenylpropanoid biosynthetic pathway, anthocyanins represent a category of pigmented secondary metabolites responsible for the rich colors of fruits, flowers, and vegetables. They are also recognized for their beneficial properties, including antioxidant and anti-inflammatory activities, and applications in the food industry and horticulture [19,20]. By applying dsRNAs targeting the chalcone synthase *AtCHS*, a specific anthocyanin biosynthesis gene, or negative regulatory genes directly to the plant surface, we observed a significant decrease or increase in the anthocyanin pigment production in *A. thaliana*. This highlights the potential of exogenous exo-RNAi for manipulating plant pigment production and potentially other plant traits. 

In addition to the degradation of mRNA, RNAi has also been found to trigger epigenetic modifications, such as alterations in DNA methylation, histone modifications, or microRNA (miRNA)-mediated processes [21,22]. These modifications can persist for a certain period of time after the initial RNAi event, potentially leading to unintended consequences. Consideration of potential unintended consequences and the specificity of gene silencing is crucial. Studies have demonstrated the ability of external dsRNA to silence genes; however, the comprehensive effects, especially on the complex system of miRNAs, have yet to be fully investigated. miRNAs are short, noncoding RNAs that are encoded in the genome and bind to target mRNAs to regulate gene expression by controlling mRNA translation and decay [23,24]. miRNAs can also be regulated by epigenetic modifications, creating a feedback loop between miRNAs and the epigenetic pathway. 

In this study, we aimed to investigate the effect of external *AtCHS*-specific dsRNAs on the miRNA transcriptome in *A. thaliana*. We found that the exogenous application of *AtCHS*-dsRNA to the *A. thaliana* leaves induced significant changes in the miRNA transcriptome. These findings have profound implications for our understanding of RNAi-mediated gene silencing in plants and highlight the need to analyze the potential epigenetic effects of external dsRNA application.

## 2. Results

### 2.1. The Analysis of Small RNA Fraction in dsRNA-Treated Arabidopsis Plants

To investigate the effects of exogenous dsRNA on the miRNA population, we analyzed six RNA-sequencing (RNA-seq) libraries generated in our previous study using the Illumina NovaSeq 6000 platform [25]. These libraries included two from water-treated plants, two from plants treated with dsRNA specific to the chalcone synthase *AtCHS* gene of *A. thaliana* (ds*CHS*), and two from plants treated with dsRNA specific to *NPTII*, a non-related bacterial neomycin phosphotransferase II gene (ds*NPTII*). The *NPTII*-dsRNA was synthesized as a control to determine whether any observed effect of exogenous dsRNA on the miRNA profiles or other effects were sequence-specific. For each plant, 100 µL of dsRNAs (equivalent to 35 µg) or 100 µL of sterile water was spread onto the leaf surfaces of four-week-old *A. thaliana* rosettes, ensuring coverage of both the upper and lower leaf areas with sterile brushes. 

Following the dsRNA treatments, the *A. thaliana* rosettes were incubated under conditions that promote *AtCHS* expression and anthocyanin production (7 °C with 23 h of light exposure) for a duration of two days. Two days after the dsRNA treatments, RNA fractions were isolated from the experimental plants. Low-molecular-weight RNA was used for the small RNA sequencing using Illumina technology [25]. High-molecular-weight RNA was used for the cDNA synthesis and quantitative real-time PCR (qRT-PCR) analysis of the miRNA target gene expression. 

**Figure 1:**
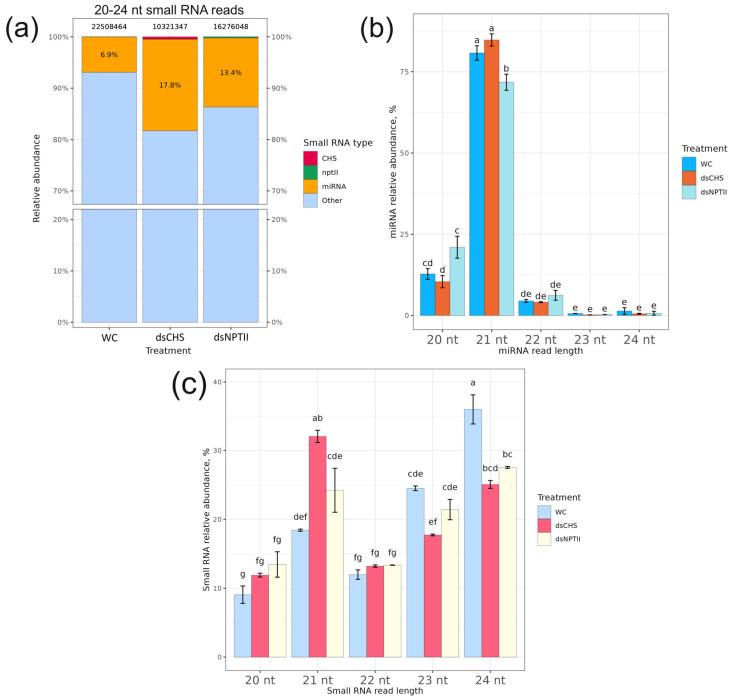
Effect of exogenous dsRNA on miRNA abundance and length distribution. (**a**) The ratio of small RNAs of 20–24 nucleotides in length in plants after water or dsRNA treatments. (**b**) Length distribution of miRNAs of 20–24 nucleotides after dsRNA and water treatments (100%—total read number of all 20–24 nt miRNAs under each treatment). (**c**) Length distribution and relative abundance of all small RNAs of 20–24 nt in length. WC—*Arabidopsis thaliana* plants treated with sterile water (two plants); ds*CHS*—*A. thaliana* treated with *AtCHS*-coding dsRNA (two plants); ds*NPTII*—*A. thaliana* treated with *NPTII*-coding dsRNA (two plants). The main classes of analyzed small RNAs are miRNAs, *AtCHS*-encoding small RNAs (*CHS*), *NPTII*-encoding small RNAs (*nptII*), and other small RNAs. Means followed by the same letter were not different using one-way analysis of variance (ANOVA), followed by the Tukey HSD multiple comparison test (*p* < 0.05).

After processing and filtering the raw sequencing reads to remove low-quality and irrelevant sequences (adapters, reads shorter than 20 and longer than 24 nucleotides, nongenomic reads (except for *NPTII*)), we obtained 4.4–12.8 million clean reads (Table S1). These reads were aligned to the *A. thaliana* genome to identify and quantify individual miRNAs. We analyzed the proportion and composition of *A. thaliana* miRNAs across different read lengths ranging from 20 to 24 nucleotides and also examined the small RNAs aligned to the *CHS* and *NPTII* sequences (Figure 1). 

The analysis of the *A. thaliana* genome identified a total of 428 miRNAs (Table S2). For the miRNA profile analysis, we examined whether exogenous *AtCHS*-dsRNA and *NPTII*-dsRNA influenced the total 20–24 nt miRNA levels within the small RNA pool (Figure 1a and Figure S1). The data revealed that the proportion of miRNAs was higher after application of the *AtCHS*-dsRNA than after treatment with the water and *NPTII* controls, reaching 17.8% in relative abundance (Figure 1a). The miRNA length size distribution analysis revealed that the prevalent miRNA lengths were 20, 21, and 22 nt miRNAs, with 21 nt miRNAs being the dominant size class, reaching 72–81% of all miRNAs (Figure 1b). The data also showed that the ds*CHS* and ds*NPTII* treatments considerably downregulated all 24 nt small RNAs compared to the water treatment (Figure 1c). It is important to note that the 24 nt small RNAs constitute the dominant small RNA class in the water control probes, mostly excluding miRNAs (Figure 1c,b). Therefore, the decrease in the 24 nt small RNA class (Figure 1c) after the dsRNA treatments contributed to the elevated proportion of miRNAs up to 17.8% and 13.4% (Figure 1a). The analysis also showed that 0.81% of 20–24 nt reads obtained after preprocessing from ds*CHS*-treated *A. thaliana* contained *AtCHS*-specific small RNAs, and 0.41% contained *NPTII*-specific small RNAs in the ds*NPTII*-treated *A. thaliana* among all the obtained 20–24 nt reads after preprocessing (Figure 1a). In contrast, no such sequences were found in the reads from the water-treated control plants.

### 2.2. The Differential Expression Profile of miRNAs in Response to Exogenous dsRNAs

**Figure 2:**
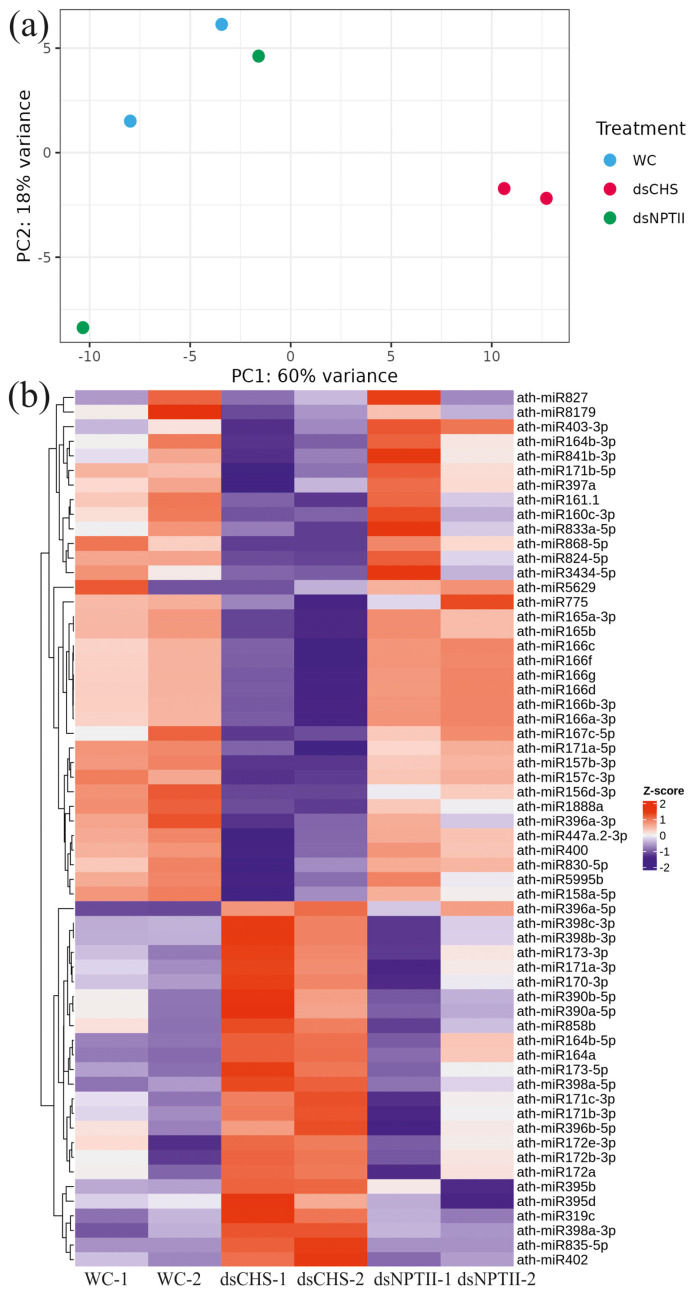
Effect of exogenous dsRNAs on miRNA profile in the *Arabidopsis thaliana* plants. (**a**) Principal component analysis (PCA) of miRNAs differentially expressed in *A. thaliana*. PCA shows the variance of two replicates per treatment x three treatments, including the water, *AtCHS*-dsRNA, and *NPTII*-dsRNA treatments. Each dot represents a sample, and each color represents a treatment. The percentage on each axis indicates the degree of variation explained by the principal components. (**b**) Heatmap showing Z-score-transformed expression levels of miRNAs that are differentially expressed in treated plants. Each row represents an miRNA, and each column represents a sample. WC-1, WC-2—two *A. thaliana* plants treated with sterile water; ds*CHS*-1, ds*CHS*-2—two *A. thaliana* plants treated with *AtCHS*-dsRNA; ds*NPTII*-1, ds*NPTII*—two *A. thaliana* plants treated with *NPTII*-dsRNA. The color intensity from red to blue indicates up- and downregulation, respectively.

We used the following analysis to determine the effect of the exogenous dsRNAs on the miRNA population two days after treatment with dsRNA or water. Using a cutoff threshold of an adjusted *p* < 0.05, we identified 60 miRNAs (14% of all detected miRNAs) that exhibited significant differential expression for ds*CHS*-treated plants or both types of dsRNA treatments vs. the water control (Figure 2; Tables S3 and S4). These differentially expressed miRNAs can be categorized into two primary groups. In the first group, 35 miRNAs were downregulated upon the *AtCHS*-dsRNA application in comparison to the water or *NPTII*-dsRNA applications (Figure 2b, Table S3). In the second group, 24 miRNAs were, in turn, upregulated upon the *AtCHS*-dsRNA application in comparison to the water control and ds*NPTII*-treated plants (Figure 2b, Table S4). We also identified ath-miR396a-5p, the level of which was significantly upregulated (*p* < 0.05) in both the ds*CHS* and ds*NPTII* treatments compared to the water treatment (Table S5). Thus, the total number of upregulated miRNAs reached 25 miRNAs. The activation of ath-miRNA396a-5p under the dsRNA treatments indicates the non-sequence-specific dsRNA effect.

A principal component analysis (PCA) of the miRNA profiles indicated that exogenous *AtCHS*-dsRNA was the primary factor affecting the overall miRNA expression in the leaves of *A. thaliana* (Figure 2a). Principal component 1 (PC1), accounting for 60% of the total variance, distinctly differentiated ds*CHS*-treated plants from the water and ds*NPTII* treatments (Figure 2a). While typically miRNAs downregulate target gene transcription via the control of mRNA translation or mRNA decay [23,24], our analysis revealed a higher number of underexpressed miRNAs after the *AtCHS*-dsRNA application. This suggests that the predominant secondary effect of gene-specific dsRNA application may be the alleviation of the silencing of the expression of certain target plant genes.

### 2.3. Target Prediction for dsRNA-Affected miRNAs and Gene Enrichment Analysis

miRNAs play a crucial role in post-transcriptional gene regulation [23,24], and their ability to target multiple transcripts simultaneously, or, in turn, the ability of a single transcript to bear several miRNA target sites, highlight the complexity of miRNA-mediated regulation. To elucidate the potential functions of miRNAs affected in response to exogenous dsRNAs, computational target prediction and gene enrichment analysis could provide valuable insights. 

Candidate mRNA targets for the differentially expressed miRNAs observed in *A. thaliana* under exogenous dsRNA treatments were predicted with the TarDB database using sequence complementarity (Tables S6 and S7). The analysis revealed that the 35 miRNAs underexpressed only after the *AtCHS*-dsRNA application (Table S3) had 282 predicted mRNA targets (Table S6), while the 24 upregulated miRNAs (Table S4) exhibited 582 predicted mRNA targets (Table S7). These target gene mRNAs represent potential candidates for miRNA-mediated regulation in response to dsRNA treatment. We also identified targets of ath-miR396a-5p, the expression of which was significantly upregulated (*p* < 0.05) in both the ds*CHS* and ds*NPTII* treatments compared to the water treatment (Table S8). 

**Figure 3:**
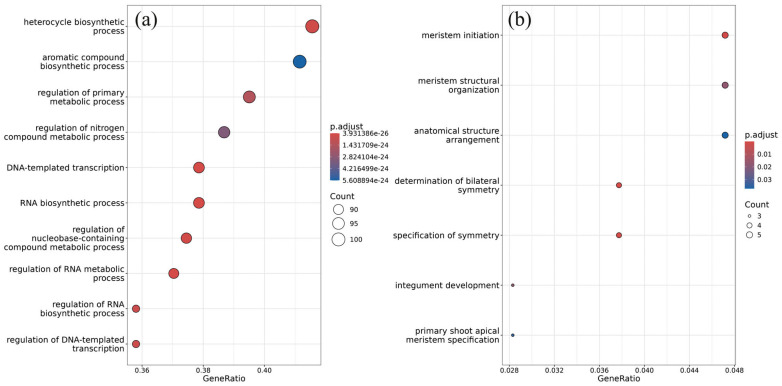
Gene set functional enrichment analysis performed with predicted targets of differentially expressed miRNAs. (**a**) Target genes of the upregulated miRNAs after the *AtCHS*-dsRNA treatment; (**b**) Target genes of the downregulated miRNAs after the *AtCHS*-dsRNA treatment. The vertical axis shows the biological processes and molecular functions of the top 10 enriched GO terms. The horizontal axis represents the gene ratio, which is defined as the ratio of the targets of differentially expressed miRNAs in a pathway to the total number of genes. The different bubble sizes represent the number of genes for that pathway. The different colors of the bubbles represent the *p*-values. The dot plots were generated using the enrichplot R package. GO analysis was performed using the gprofiler2 R package.

Gene Ontology (GO) analysis is a widely used technique to uncover the putative biological functions of genes [26]. By categorizing target genes based on their functional annotations, GO analysis could aid in understanding the overall impact of miRNA regulation in response to external dsRNA. Enrichment analysis of miRNA targets is a common approach to uncovering the hierarchical activities of miRNAs in gene regulation networks [27]. Using the R package gprofiler2 (version 0.2.3), GO-based enrichment analysis was performed on the mRNA targets of the differentially expressed miRNAs (Figure 3a,b). Gene list functional enrichment analysis of the targets of the 25 dsRNA-upregulated miRNAs (Figure 3a) showed the enrichment of processes associated with aromatic and heterocycle biosynthetic processes, the regulation of primary metabolism, DNA-templated transcription, and RNA biosynthesis and RNA metabolism; for a complete list of terms, see Tables S9 and S11. Notably, these processes were inferred to be downregulated by the miRNA-mediated regulation. This finding suggests that the 25 upregulated miRNAs may contribute to the suppression of these biological pathways.

At the same time, gene list functional enrichment analysis of the genes targeted by the 35 ds*CHS*-downregulated miRNAs showed the enrichment of development-related processes involved in meristem initiation, organization, and specification (Figure 3b; Table S10). It is possible that these processes were upregulated in response to the *AtCHS*-dsRNA treatment. However, the support of the gene overrepresentation of the downregulated miRNAs was considerably lower than that of the upregulated miRNAs.

### 2.4. Validation of miRNA Target Gene Data by qRT-PCR

**Figure 4:**
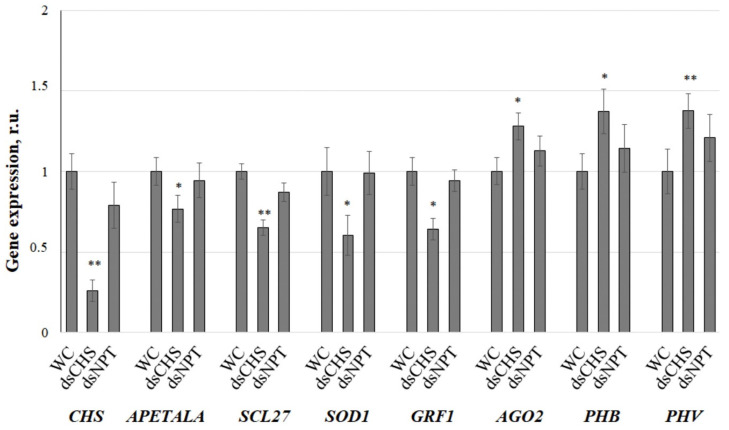
RT-qPCR validation of differentially expressed microRNA target genes under dsRNA treatments and control samples. The effects of external *AtCHS*- and *NPTII*-encoding dsRNAs on miRNA target genes and *AtCHS* mRNA level in *Arabidopsis thaliana* were analyzed by quantitative real-time PCR. *A. thaliana* plants were grown under anthocyanin-inducing (7 °C, 23 h light) conditions for two days after treatment with sterile water or synthetic dsRNA. qRT-PCR data are presented as the mean ± SE. *, **—significantly different from WC at *p* ≤ 0.01 according to Student’s *t*-test.

By qRT-PCR analysis, we confirmed that the application of *AtCHS*-dsRNA to the *A. thaliana* leaves resulted in a significant 4-fold reduction in the *AtCHS* mRNA levels compared to the water treatment (Figure 4). Conversely, the unrelated *NPTII*-dsRNA showed no significant effect on the *AtCHS* expression. Then, the investigation turned to validating several predicted targets of seven distinct miRNA families either ds*CHS*-upregulated (ath-miR171, ath-miR172, ath-miR398, ath-miR396) or ds*CHS*-downregulated (ath-miR166, ath-miR165, ath-miR403) in comparison with the water control and ds*NPTII* application. The selected miRNAs were chosen due to their significant differential expression between the ds*CHS*-treated plants vs. the water and ds*NPTII* treatments (Figure 2; Tables S3 and S4) and the established roles of their corresponding mRNA targets [28,29,30,31,32]. Their respective mRNA targets, including *APETALA2*, *SCL27*, *SOD1*, *GRF1*, *AGO2*, *PHB*, and *PHV*, are associated with diverse biological functions ranging from plant development to stress responses. 

To verify the miRNA-target interactions, we measured the expression levels of the chosen target genes in the treated *A. thaliana* by qRT-PCR (Figure 4). It is known that miRNAs negatively regulate gene expression either by inhibiting mRNA translation or promoting RNA degradation [23,24]. Therefore, the expected outcome is a negative correlation between the expression of a specific miRNA and its corresponding target mRNA. In other words, downregulation of an miRNA should lead to the increased expression of its target gene, while upregulation of an miRNA should result in the decreased expression of its target gene. 

The qRT-PCR analysis reveals a strong negative correlation in the expression of the pairs of miRNAs and their predicted targets (Figure 2 and Figure 4). For example, three closely related miR172 family members, ath-miR172a, ath-miR172b-3p, and ath-miR172e-3p, were upregulated after treatment with *AtCHS*-dsRNA (Figure 2), while the expression of their target—a floral homeotic *APETALA2* gene—decreased in comparison with the water treatment and *NPTII* controls (Figure 4). Similarly, closely related family members ath-miR171a-3p/ath-miR171b-3p/ath-miR171c-3p, ath-miR398b-3p/ath-miR398c-3p, and ath-miR396b-5p were upregulated after the *AtCHS*-dsRNA treatment (Figure 2), while the expression of their respective targets—GRAS family transcription factor SCL27, the superoxide dismutase *SOD1* gene, and growth regulating factor *GRF1* genes—was downregulated (Figure 4). Conversely, while the ath-miR403-3p and seven ath-miR166 family members were downregulated by *AtCHS*-dsRNA, their target genes *AGO2*, *PHB*, and *PHV* were activated (Figure 4).

## 3. Discussion

The potential of using exogenous dsRNA to manipulate gene expression in plants and pathogens is gaining significant attention. This innovative approach, known as exo-RNAi or SIGS, offers a promising alternative to traditional crop management methods, providing environmentally friendly and low-risk solutions for crop trait tailoring [1,2,3,4,5]. Recently, we showed that applying gene-specific dsRNAs and siRNAs to the leaves of *A. thaliana* considerably diminished the expression of the target genes implicated in anthocyanin biosynthesis regulation, including *AtCHS* and five repressors of anthocyanin biosynthesis [11,13]. This reduction in *AtCHS* expression, a key enzyme in the anthocyanin pathway, was accompanied by the emergence of specific small RNAs against *AtCHS*, indicating the involvement of RNAi in the plant response to the exogenous dsRNA [25]. Furthermore, a study revealed that exogenous *AtCHS*-dsRNA not only penetrates individual plant cells but also travels through the vascular system, reaching various parts of the plant. This systemic movement suggests the wide-reaching potential for dsRNA-based treatments [11].

Several other studies have shown that exogenous dsRNAs applied to plant leaves are capable of regulating plant endogenes [9,10,12,14,15], yet little is known about the exogenous plant dsRNA treatment impacts on plant epigenetics, including miRNA profiles. In this study, we found that the exogenous application of *AtCHS*-dsRNA to the leaves of *A. thaliana* induced extensive changes in the miRNA transcriptome, considerably affecting the expression of 59 miRNAs, while the non-sequence-specific bacterial *NPTII*-dsRNA had a low effect, affecting the expression of only 1 miRNA. The miRNA profile after the *AtCHS*-dsRNA application was considerably different from that after the control treatments. First of all, this finding revealed that gene-specific dsRNAs induce specific and massive cellular responses at the epigenetic level after exogenous application. Thus, *AtCHS*-dsRNA induced not only specific *AtCHS* gene downregulation but also extensive changes in the epigenetic environment after treatment. Recently, exogenous dsRNA targeting a plant promoter sequence was shown to induce the de novo methylation of promoter sequences in tobacco plants [33]. To the best of our knowledge, no other reports on the epigenetic environment after dsRNA plant treatments have been published. The dsRNA-induced miRNA alterations that are presumably the result of the inhibited *AtCHS* gene expression suggest that the *AtCHS* gene is tightly epigenetically regulated by miRNAs. It is possible that our treatment disrupts this miRNA-mediated feedback.

The qRT-PCR analysis revealed a strong negative correlation between the expression of miRNAs and their predicted targets. This compelling evidence solidifies the validity of these predicted interactions, providing valuable insight into the intricate regulatory networks orchestrated by these miRNA families. For example, ath-miR403 was downregulated after the *AtCHS*-dsRNA application, and verification confirmed the increased gene expression of its target *AGO2*, which is the key effector component of the RNAi machinery [34]. This indicates an activated RNAi system in response to exogenous gene-specific dsRNA application. 

The miR171-SCL and miR396 modules are involved in the regulation of chlorophyll biosynthesis and leaf growth in *A. thaliana* [29,30]. This study showed that three closely related miR171 family members (ath-miR171a-3p, ath-miR171b-3p, and ath-miR171c-3p) were upregulated after the *AtCHS*-dsRNA application, while the expression of the target gene *SCL27* was inhibited. The data suggest that the ds*CHS* treatment had a negative effect on chlorophyll biosynthesis and new organ formation in the *A. thaliana*. SOD1 is implicated in the regulation of plant stress resistance and immune reactions [32]. Activation of ath-miR398b-3p, ath-miR398b-3p, and ath-miR398c-3 was associated with the downregulation of the *SOD1* target gene. Thus, it is possible that the exogenous dsRNA compromises plant stress resistance mechanisms. Downregulated miR165 and miR166 levels correlated with the increased expression of the *PHB* and *PHV* genes, suggesting the positive effect of exogenous *AtCHS*-dsRNA on shoot apical meristem and floral development, which are regulated by miR166/165. Thus, this study indicates that exogenous dsRNA activates RNAi-mediated processes, meristem formation, and flowering, and it downregulates new plant organ formation and stress tolerance.

## 4. Methods and Materials

### 4.1. Plant Material and Growth Conditions

The seeds of wild-type *Arabidopsis thaliana* from the Columbia (Col-0) ecotype underwent a sterilization process using vapor-phase sterilization. Then, the seeds were sown on solid ½ Murashige and Skoog (MS) medium as described [25]. One week later, the germinated seedlings were planted in 7 cm × 7 cm pots filled with 100 g of commercially available, rich soil. The plants were cultivated in a controlled environmental chamber at 22 °C under plastic wrap for an additional three weeks without extra watering [25]. To induce anthocyanin production and allow for the evaluation of the gene silencing efficiency after the dsRNA treatment, the four-week-old plants were incubated without further irrigation for an additional two days in a growth chamber (KS-200, Smolenskoe SKTB SPU, Smolensk, Russia) with a temperature of 7 °C and daily light period of 23 h before RNA isolation.

### 4.2. dsRNA Synthesis

The T7 RiboMAX™ Express RNAi System (Promega, Madison, WI, USA) was employed as described to generate *AtCHS*-dsRNA and *NPTII*-dsRNA [25]. For this purpose, a large cDNA fragment of *AtCHS* (AT5G13930.1, 736 bp out of 1188 bp) was amplified via PCR for in vitro transcription and dsRNA synthesis. Additionally, a significant portion of *NPTII* (GenBank AJ414108, 599 bp out of 798 bp) was amplified using the pZP-RCS2-nptII plasmid [35]. In a single PCR for each gene, the T7 promoter sequence was added to both the 5′ and 3′ ends of the amplified *AtCHS* or *NPTII* as detailed [25]. These PCR fragments were then run on a 1% agarose gel for separation and subsequently purified. The purified PCR products served as templates for the in vitro transcription and dsRNA synthesis according to the manufacturer’s instructions. The resulting dsRNAs were assessed through gel electrophoresis and spectrophotometry to determine their purity, integrity, and quantity.

### 4.3. Plant dsRNA Treatment

For the dsRNA application, four-week-old wild-type *A. thaliana* rosettes were treated by applying *AtCHS*- and *NPTII*-dsRNAs to the leaf surfaces with individual sterilized soft brushes made from natural pony hair, which had been sterilized in an autoclave [11,36]. Each treatment involved diluting 35 µg of the dsRNAs in 100 µL of nuclease-free water and covering all rosette leaves on both the upper and lower surfaces with the mixture [25]. Two rosettes per treatment condition were treated, with 35 µg of dsRNA administered to each rosette. Plant dsRNA treatments were performed during late evening hours (21:00–21:30) under conditions of low soil moisture [25]. This method of dsRNA application has been shown to be effective in recent studies [11,36].

### 4.4. RNA Isolation

To extract RNA from samples, we collected two whole four-week-old rosettes of *A. thaliana* two days post-treatments: water treatment (rosettes WC-1, WC-2), *AtCHS*-dsRNA treatment (rosettes ds*CHS*-1, ds*CHS*-2), and *NPTII*-dsRNA treatment (rosettes ds*NPTII*-1, ds*NPTII*-2). The RNA isolation process involved separating high-molecular-weight and low-molecular-weight RNA fractions by a modified protocol based on cetyltrimethylammonium bromide (CTAB) and lithium chloride (LiCl) application as described [25]. Initially, total RNA extraction was initiated using the CTAB–polyvinylpyrrolidone K30 (PVP) extraction buffer prepared as detailed [37]. Subsequently, high-molecular-weight RNAs were isolated by treating the pellets as described [25]. The pellets after air drying were resuspended in sterile, filtered water and utilized for complementary DNA (cDNA) synthesis. To obtain the low-molecular-weight RNA fraction, after precipitating with LiCl, 400 µL of the subsequent upper aqueous layer was moved to a new, clean 1.5 mL collection tube and precipitated [25]. 

### 4.5. Reverse Transcription and qRT-PCRs

Synthesis of cDNAs was performed with 1.5 µg of total RNA according to the procedure described [37]. To ensure the absence of DNA contamination, 1 µL aliquots of reverse transcription products were subjected to PCR amplification with specific primers for the *AtCHS* gene (Table S9). Reverse transcription and the qRT-PCRs were conducted using SYBR Green I Real-Time PCR dye and a real-time PCR kit (Evrogen, Moscow, Russia), as described [38], with *UBQ* and *GAPDH* serving as internal controls. These genes were previously identified as appropriate reference genes for qRT-PCRs in Arabidopsis [39]. A DTprime real-time thermocycler (DNA Technology, Moscow, Russia) was used for amplification with an initial step of 2 min at 95 °C, followed by 50 cycles at 95 °C for 10 s and at 62 °C for 25 s. The calculation of the expression was performed using the 2^−ΔΔCT^ method [40]. The data analysis was carried out utilizing RealTime_PCR version 7.3 (DNA Technology, Russia). Table S12 contains all gene identification numbers along with the primers employed.

### 4.6. Sequencing of Small RNAs

The RNA fractions with low molecular weights, isolated through ethanol precipitation, were sent to Evrogen in Moscow, Russia, for high-throughput sequencing utilizing Illumina technology [25]. Quality control of the incoming samples, RNA preparation, and small RNA sequencing were conducted as previously described [25]. After performing quality control and measuring the DNA concentration, the library collection was sequenced using an Illumina NovaSeq 6000 system with single-read sequencing at 100 bp according to the established protocol [25]. This process yielded a total of 526,174,120 reads. An overview of the read numbers is provided in Table S1. The sequences from the sRNA library have been deposited in the National Center for Biotechnology Information (NCBI) with Accession Number PRJNA827691 and are also accessible in the database of the Laboratory of Biotechnology at the Federal Scientific Center of East Asia Terrestrial Biodiversity, Far Eastern Branch of the Russian Academy of Sciences, Russia (https://biosoil.ru/downloads/biotech/RNAseq/Arabidopsis/2021-03-20012551-data1(Our-RNAseq-1(2))/ (accessed on 4 July 2024)).

### 4.7. Small RNA Identification and Analysis

Adaptor sequences (5′AACTGTAGGCACCATCAAT, 5′AGATCGGAAGAGCACACGT) were cut off, and reads of poor quality as well as those shorter than 20 nucleotides and longer than 24 nucleotides were filtered out from the obtained high-throughput sequencing data using the BBDuk program (version 39.06) [41]. Using the Bowtie program (version 1.3.1), allowing 0 mismatches [42], reads aligned to the *A. thaliana* TAIR10.1 genome [43] (NCBI Accession Number GCF_000001735.4) with the added *NPTII* gene and unaligned reads were excluded from analysis. The obtained SAM format files were converted into BAM files and indexed using SAMtools (version 1.20) [44]. The processing of the data was performed using the R programming language. Using the GenomicAlignments R package (version 1.38.0) [45], we analyzed the small RNAs aligned to *AtCHS* and *NPTII* sequences and miRNAs obtained from the TAIR10.1 genome (Table S1) with miRBase accession IDs [46]. *AtCHS*, *NPTII*, and miRNA reads were counted using the summarizeOverlaps function from the GenomicAlignments R package. The bar plots of the relative abundance of small RNA fractions were plotted using the ggplot2 (version 3.5.0) [47] and ggbreak (version 0.1.2) [48] R packages based on 20–24 nt reads. 

### 4.8. Differential Expression Analysis of miRNAs

Using DESeq2 (version 1.44.0) [49], we analyzed differentially expressed miRNAs for the ds*CHS* treatment compared to the water and ds*NPTII* controls, and for the ds*CHS* and ds*NPTII* treatments compared to the water control. We identified differentially expressed miRNAs according to the standard algorithm [50,51,52,53]. For the differential miRNA expression analysis, we used counts of reads aligned to Arabidopsis miRNAs as input for the DESeq2 tool [49]. MiRNA count data were transformed using the variance-stabilizing transformation method and visualized on a PCA ordination plot using the vst and plotPCA functions of the DESeq2 R package, respectively. Differentially expressed miRNA counts were visualized on a heatmap using the ComplexHeatmap R package (version 2.20.0) [54].

### 4.9. Target Gene Prediction and Functional Annotation

The target genes of differentially expressed miRNAs were obtained using the TarDB database [55]. Gene set enrichment analysis (GSEA) of target genes was performed using the gProfiler2 R package (version 0.2.3) [56] across the Gene Ontology–Biological Process (GO–BP) database [26], and the results were visualized using the enrichplot R package (version 1.24.2) [57].

### 4.10. Statistical Analysis

The distribution data of miRNAs, which are 20–24 nucleotides long, are shown as the mean ± standard error (SE) and were analyzed using a one-way ANOVA, followed by the Tukey HSD test for multiple comparisons. In the DESeq2 analysis, *p*-values were corrected for multiple testing using the Benjamini–Hochberg procedure. In the GSEA, the default *p*-value correction method g:SCS was used. The RT-qPCR validation data are presented as the mean ± SE and were evaluated using a paired Student’s *t*-test. A *p*-value of less than 0.05 was determined as the threshold for statistical significance in all the tests. For each treatment type, two whole rosettes of *A. thaliana*, four weeks old, were collected two days post-treatment (yielding two biological replicates for each type of treatment).

## 5. Conclusions

This study found that the external *AtCHS*-specific dsRNAs had a profound effect on the miRNA transcriptome in *A. thaliana*, inducing significant changes in the miRNA composition and affecting the expression of the miRNA target genes. These findings provide new insights into the complex interplay between dsRNA treatments and miRNA expression in plants. Further research is needed to determine whether external dsRNAs can trigger epigenetic alterations that persist over time, and to assess the potential risks and benefits of using exo-RNAi approaches. In addition, this study sheds light on the delicate interplay between miRNAs and their target genes, highlighting their crucial role in shaping the complex biological processes in *A. thaliana*.

## Data Availability

The data presented in this study are available within the article and Supplementary Materials.

## Supplementary Materials

The following supporting information can be downloaded at https://www.mdpi.com/article/10.3390/plants13162335/s1, Figure S1. 10–100 nt RNAs obtained after high-throughput sequencing and aligned on the Arabidopsis genome. Table S1. Samples analyzed by sRNA-seq and read numbers of 20–24 nt small RNAs obtained after high-throughput sequencing (Illumina NovaSeq 6000 instrument). Table S2. MicroRNAs in *A. thaliana* TAIR10.1 genome. Table S3. Significantly downregulated microRNAs after dsCHS treatment compared to water and dsNPTII treatment (adjusted *p* < 0.05). Table S4. Significantly upregulated microRNAs after dsCHS treatment compared to water and dsNPTII treatment (adjusted *p* < 0.05). Table S5. Significantly upregulated microRNAs after both dsCHS and dsNPTII dsRNA treatments compared to water treatment (adjusted *p* < 0.05). Table S6. Targets of significantly downregulated microRNAs after dsCHS treatment compared to water and dsNPTII treatment according to the TarDB database. Table S7. Targets of significantly upregulated microRNAs after dsCHS treatment compared to water and dsNPTII treatment according to the TarDB database. Table S8. Targets of significantly upregulated microRNAs after both dsCHS and dsNPTII dsRNA treatments compared to water treatment according to the TarDB database. Table S9. List of significantly enriched functions in predicted mRNA targets of dsCHS-upregulated miRNAs. Table S10. List of significantly enriched functions in predicted mRNA targets of dsCHS-downregulated miRNAs. Table S11. Targets of significantly upregulated microRNAs after both dsCHS and dsNPTII dsRNA treatments compared to water treatment according to the TarDB database. Table S12. Primers used in RT-PCR and qRT-PCRs.
