# Supplementary material for: Impact of Exogenous dsRNA on miRNA Composition in Arabidopsis thaliana
Source: Plants (Basel). 2024 Aug 22;13(16):2335. doi: 10.3390/plants13162335 (PMC11360658; doi:10.3390/plants13162335)
Supplement: Supplementary file 1 [file plants-13-02335-s001.zip › Table S12.pdf]

Supplementary Table S12. Primers used in RT-PCR and qRT-PCRs.

Gene name (ID number)	Primer name	Primers, 5′-3′
Primers for PCR and real-time PCR, 5′-3′		
AtCHS (AT5G13930.1)	AtCHS-Nach-S<br>AtCHS-realA	5′ATGGTGATGGCTGGTGCTTCTT<br>5′- CACATGGTTCTCAGGGTTAGC
AtAPETALA2 (AT4G36920.1)	RT-APETALA2-s:<br>RT-APETALA2-a:	GGCCTCGACGAACCAAGTGT<br>TCATGAGAGGAGGTTGGAAGCC
AtSCL27 (AT2G45160.1)	RT-SLP27-s:<br>RT-SLP27-a:	CACCGGAAACAACAACGGCG<br>GAGGAGAGCTTCAGCGATGTGA
AtSOD1 (AT1G08830.1)	RT-CSD1-s:<br>RT-CSD1-a:	GCTTTTGGACCTCGTGGGCTAT<br>GTCCCCGTAACACCCTCACT
AtGRF1 (AT2G22840.1)	RT-GRF1-s:<br>RT-GRF1-a:	GGGACGCTGTTCCCGATCAA<br>CACGCATCGCAACAGCTGAA
AtAGO2 (AT1G31280.1)	RT-AGO2-s:<br>RT-AGO2-a:	TTCGAAATCGGAACGCCCCAA<br>ACCCAATTAGATTCACGCACAAACT
AtPHB *(AT2G34710.1)*	RT-PHB-s:<br>RT-PHAB-a:	ACAACCCAGCAGGACTCCTTT<br>TGCGCGAAATAGCGACTATGC
AtPHV (AT1G30490.1)	RT-PHAV-s:<br>RT-PHAV-a:	TCTCGATTGCGGAGGAGACC<br>CACGTGCTGCTATTCCACTGC
AtGAPDH (GenBank NM_111283)	AtGapdh-real-s,<br>AtGapdh-real-a	5′TTG GTG ACA ACA GGT CAA GCA,<br>5′AAA CTT GTC GCT CAA TGC AAT
AtUBQ (GenBank NM_001084884)	AtUBQ-realS,<br>AtUBQ-realA	5′GGCCTTGTATAATCCCTGATGAATAAG,<br>5′AAAGAGATAACAGGAACGGAAACATAGT
Specific primers for dsRNA design, 5′-3′		
AtCHS (AT5G13930.1)	AtCHS-RNAs2<br>AtCHS- RNA-a2	5'TAATACGACTCACTATAGGGAGAGCTTCTTGGTCTCC GTCCTTCC<br>5'TAATACGACTCACTATAGGGAGATTAGAGAGGAACG CTGTGCAAG
NPTII (AY818371)	npt-T71-s,<br>npt-T72-a	5′TAATACGACTCACTATAGGGAGAATGTGGATTGAAC AAGATGGATTG,<br>5′TAATACGACTCACTATAGGGAGATCCACCATGATATT CGGCAAGCAG
Primers for cDNA check-up on DNA contamination, 5′-3′		
AtGAPDH (GenBank NM_111283)	AtGapdh-s,<br>AtGapdh-a	5′CTG GAA TGT CTT TCC GTG TC,<br>5′ATT CGT TGT CGT ACC ATG AC
